# Reduced regadenoson dose using heart rate-targeted titration in paediatric stress perfusion CMR

**DOI:** 10.1093/ehjimp/qyag108

**Published:** 2026-06-15

**Authors:** Michael A Quail, Oliver Tann, Romina Linton, Elena G Milano, Kristian Mortensen, Andrew M Taylor, Vivek Muthurangu

**Affiliations:** Centre for Cardiovascular Imaging, Heart and Lung Division, Great Ormond Street Hospital for Children NHS Foundation Trust, Great Ormond Street, London WC1N 3JH, UK; Research Department of Children’s Cardiovascular Disease, Institute of Cardiovascular Science, University College London, London WC1N 1DZ, UK; Centre for Cardiovascular Imaging, Heart and Lung Division, Great Ormond Street Hospital for Children NHS Foundation Trust, Great Ormond Street, London WC1N 3JH, UK; Centre for Cardiovascular Imaging, Heart and Lung Division, Great Ormond Street Hospital for Children NHS Foundation Trust, Great Ormond Street, London WC1N 3JH, UK; Centre for Cardiovascular Imaging, Heart and Lung Division, Great Ormond Street Hospital for Children NHS Foundation Trust, Great Ormond Street, London WC1N 3JH, UK; Centre for Cardiovascular Imaging, Heart and Lung Division, Great Ormond Street Hospital for Children NHS Foundation Trust, Great Ormond Street, London WC1N 3JH, UK; Centre for Cardiovascular Imaging, Heart and Lung Division, Great Ormond Street Hospital for Children NHS Foundation Trust, Great Ormond Street, London WC1N 3JH, UK; Research Department of Children’s Cardiovascular Disease, Institute of Cardiovascular Science, University College London, London WC1N 1DZ, UK; Research Department of Children’s Cardiovascular Disease, Institute of Cardiovascular Science, University College London, London WC1N 1DZ, UK

**Keywords:** regadenoson, stress perfusion, cardiac magnetic resonance, paediatric cardiology, kawasaki disease, congenital heart disease, pharmacological stress testing, coronary artery disease

## Abstract

**Aims:**

Weight-based regadenoson dosing for paediatric stress perfusion cardiac magnetic resonance (CMR) achieves coronary vasodilation but may result in excessive drug exposure in some patients. We evaluated the feasibility, safety, and diagnostic performance of heart rate-targeted regadenoson titration as an individualized alternative to fixed-based dosing.

**Methods and results:**

We performed a retrospective analysis of 31 consecutive regadenoson stress perfusion CMR studies (29 technically successful) in paediatric patients with known or suspected coronary artery disease (2015–2025). Regadenoson was administered incrementally, targeting ≥20% heart rate increase. Outcomes included achieved doses, haemodynamic responses, adverse events, image quality, and positivity rate. Results were compared with published weight-based dosing protocols.Mean age was 11.8 ± 4 years (range 3–18) and weight 50.4 ± 21 kg (range 14.9–91.8). Complete dosing data were available for 21/29 studies. Target heart rate response was achieved in all 29 studies (100% success) with a median dose 3.2 mcg/kg (range 1.6–8), representing 60% reduction compared with standard 8 mcg/kg protocols. Among patients >50 kg, 83% achieved adequate stress with ≤160 mcg (≤40% of the standard 400 mcg adult dose). Mean heart rate increased 42% (range 16–77%). Transient blood pressure reductions ≥10 mmHg occurred in nine patients. All studies yielded diagnostic-quality images. Stress-inducible perfusion defects were identified in 7/29 studies (24%). No major adverse events occurred.

**Conclusion:**

Heart rate-targeted regadenoson titration for paediatric stress perfusion CMR is feasible, achieving diagnostic-quality images with substantially reduced drug exposure. Consistent haemodynamic responses and positivity rates comparable to published series suggest adequacy of pharmacological stress, though prospective validation with quantitative perfusion measurement is required.

## Graphical Abstract

**Graphical Abstract:**
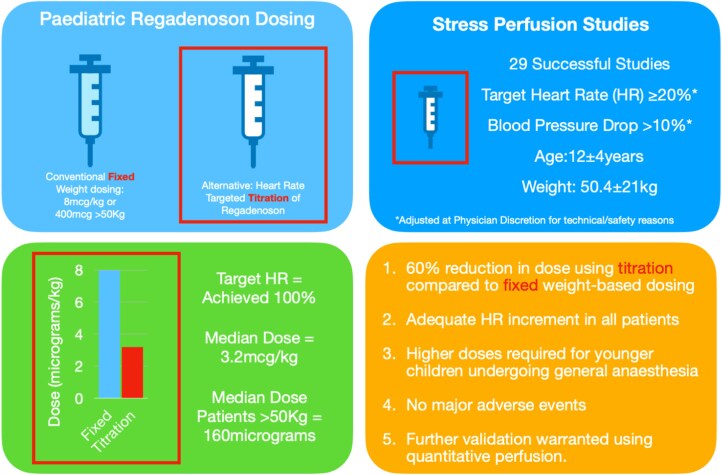

## Introduction

Stress perfusion cardiac magnetic resonance imaging (CMR) is a valuable non-invasive tool for assessing myocardial ischaemia in paediatric patients with coronary artery pathology. Although often performed using Adenosine as the stressor, regadenoson (a selective adenosine A2A receptor agonist) offers significant practical advantages. These include single bolus administration via one peripheral intravenous line, rapid onset of peak effect within 30 s, and a reduced incidence of atrioventricular conduction disturbance and bronchospasm resulting from selective A2A receptor targeting.^1,2^ Standard adenosine protocols typically require two intravenous lines (one dedicated to adenosine infusion and one for contrast administration) and require a more complex setup for continuous infusions in the MRI environment. These characteristics make regadenoson particularly attractive for paediatric imaging, where minimizing patient discomfort and ensuring procedural simplicity is desirable due to the low frequency of stress perfusion studies in most paediatric centres.

Published paediatric regadenoson protocols employ weight-based dosing strategies, typically administering 6–10 mcg/kg with a maximum dose of 400 mcg, or a standardized 8 mcg/kg dose.^3–6^ This approach is extrapolated from adult dose-escalation studies demonstrating dose-dependent coronary vasodilatation.^1,7^ Studies in children weighing less than 40 kg have confirmed the safety and feasibility of 8 mcg/kg dosing, with predominantly minor, self-limited side effects.^6^ These include flushing, dyspnoea, and transient hypotension, the latter occurring more frequently in sedated patients.

Despite the demonstrable safety of weight-based dosing, several observations suggest that individualized dosing strategies warrant further investigation. First, substantial variation in haemodynamic response to regadenoson is seen in children, with heart rate increases ranging from 14% to 145% (with greater increases associated with adverse events).^3–6,8–11^ Secondly, factors including age, baseline sympathetic tone, concurrent medications, and requirement for general anaesthesia influence drug response, yet fixed weight-based protocols treat all patients identically regardless of these variables. Finally, no paediatric dosing schedule has been validated using quantitative measures of coronary hyperaemia in paediatric populations. All current approaches rely on indirect markers or assumptions about adequate stress.

We investigated an alternative strategy: incremental titration of regadenoson to achieve a predetermined physiological response (heart rate increase ≥20% from baseline, blood pressure decrease >10 mmHg, or clinical symptoms), rather than administering a calculated weight-based dose to all patients. This approach is feasible due to the rapid pharmacological effect of regadenoson. Theoretically, titration could reduce drug exposure while maintaining diagnostic efficacy by administering only the dose necessary to achieve adequate stress markers. Additionally, by monitoring physiological response during administration, titration may avoid excessive tachycardia (>140 bpm) that necessitates sequence modifications, including reduced spatial resolution and decreased slice coverage.

In this single-centre study, we performed a retrospective analysis of a heart rate-targeted titration protocol for regadenoson in paediatric stress perfusion CMR. Our aims were to: (i) assess the feasibility and safety of this approach; (ii) describe the doses required to achieve target physiological responses; (iii) investigate factors influencing dose requirements, particularly age and general anaesthesia; (iv) assess the diagnostic yield (positivity rates) for stress-inducible perfusion defects; and (v) compare our findings with published weight-based dosing protocols to generate hypotheses for future prospective validation studies.

## Methods

All Regadenoson stress perfusion imaging studies performed at Great Ormond Street Hospital for children between 2015 and 2025 were reviewed. This was a retrospective, observational, single-centre study without concurrent controls. Informed consent for the use of imaging data was obtained from all parents or guardians of the patients included in this study. The study protocol conforms to the ethical guidelines of the 1975 Declaration of Helsinki and was approved by the local committee of the UK National Research Ethics Service (06/Q0508/124). GOSH local audit/service evaluation reference (#3290).

### General anaesthesia

The decision for general anaesthesia was made by the clinical team based on patient age, ability to cooperate with breath-holding instructions, and clinical status. When required, general anaesthesia was administered and monitored by a paediatric anaesthetist. Induction of anaesthesia was achieved using either an inhalational technique with sevoflurane or an intravenous technique with propofol, depending on the individual patient. Neuromuscular blockade was used to ensure immobility during image acquisition, with atracurium or rocuronium used for muscle relaxation. Anaesthesia was maintained in all patients with sevoflurane, titrated to expired concentrations of approximately 1.5–2.5% in an oxygen/air mixture for the duration of scanning. Antiemetic and anti-sialagogue agents were administered as indicated. Intravenous crystalloid was used if required for fluid maintenance.

### Regadenoson stress perfusion protocol: patient preparation

Patients were instructed to abstain from caffeine-containing products (coffee, tea, chocolate, caffeinated soft drinks) and medications containing theophylline or dipyridamole for 24 h prior to imaging. A 1-h fast was required immediately before the examination. Pre-procedure screening excluded patients with severe asthma or contraindications to regadenoson or gadolinium-based contrast agents. Resuscitation equipment was available for all studies.

### Imaging protocol

All studies were performed on a 1.5T MRI scanner (MAGNETOM Avanto Fit, Siemens Healthineers, Erlangen, Germany). Following standard localizer imaging, cine steady-state free precession (SSFP) sequences were acquired in left ventricular long axis, right ventricular long axis, and four-chamber views. A short-axis stack was planned, and three breath-held SSFP slices were positioned at basal, mid-ventricular, and apical levels for subsequent perfusion imaging.

### Heart rate-targeted regadenoson titration protocol

The following heart rate-targeted titration approach was established as a predefined institutional paediatric regadenoson protocol at our centre.

A single peripheral intravenous cannula (18–22G) was sited prior to imaging. Baseline heart rate and non-invasive blood pressure were recorded with continuous ECG monitoring and pulse oximetry throughout the procedure. All studies were clinically led by either MQ (paediatric cardiologist) or OT (cardiovascular radiologist) during the study period.

### Dosing regimen:

Regadenoson was administered in incremental doses via the peripheral cannula by slow IV injection. Heart rate was monitored continuously between each aliquot, with subsequent doses given only if the target heart rate increase had not been achieved, according to the following protocol:

- *Calculated dose:* 8 mcg/kg (maximum 400 mcg for patients >40 kg), prepared from stock vials containing 400 mcg in 5 mL (80 mcg/mL).
- *Administration method:* Incremental doses were administered by slow IV injection (typically 1 mL volumes), each followed by saline flush, using a three-way tap valve. Clinicians waited for 30 s after saline flush for a haemodynamic effect before administering a further incremental dose.
- *Target heart rate:* A target heart rate increase of ≥20% from baseline was defined as representing adequate pharmacological stress. However, this target could be adjusted at the discretion of the supervising clinician for the following reasons: clinical concern that an HR increase ≥20% would be associated with adverse events, or in cases of baseline tachycardia where a HR increase ≥20% was considered likely to compromise image acquisition.
- *Titration endpoint:* Drug administration ceased once the target heart rate response was achieved and remained stable for 30 s.
- *Alternative stopping criteria:* A reduction in blood pressure ≥10 mmHg or significant clinical symptoms of flushing, chest pain, breathlessness, or distress.

### First-pass perfusion imaging

Once the target heart rate was achieved, stress first-pass perfusion imaging was performed. Gadolinium-based contrast agent (0.1 mL/kg gadoterate meglumine [Dotarem, Guerbet], maximum 10 mL) was administered at 2–4 mL/s via power injector, followed by saline flush. Patients were asked to hold their breath for as long as possible, then revert to shallow respiration during the acquisition, or, for those imaged under general anaesthesia, data were acquired during end-expiratory apnoea (suspended ventilation).

Fifty to seventy dynamic perfusion images per slice were acquired at three short-axis levels (basal, mid-ventricular, and apical). Due to the sequential multi-slice acquisition scheme within a single heartbeat, the effective cardiac phase varied slightly between slices and did not correspond to a standardized systolic or diastolic time point.

During the study period two standard vendor (Siemens) perfusion sequences (without inline motion correction or artificial intelligence-based reconstruction) were used: Prior to May 2018 (*n* = 6) a saturation-recovery gradient echo sequence was used with the following parameters: typical echo time (TE) 1.0–1.2 ms, repetition time (TR) 2.3–2.5 ms, inversion time (TI) 110–120 ms, flip angle 12°, slice thickness 8–10 mm. After May 2018 (*n* = 23), a balanced SSFP sequence was used with the following parameters: typical echo time (TE) 0.95–1.01 ms, repetition time (TR) 2–3 ms, inversion time (TI) 90–120 ms, flip angle 50°, slice thickness 8–10 mm. Field of view was adjusted for patient size (typically 280–380 mm). Parallel imaging with an acceleration factor of 2 was used for both implementations. In-plane resolution was maintained at 1.8–2.5 mm to ensure adequate spatial resolution for visualization of small coronary territories in younger children. Sequence parameters including trigger delay, base resolution, and receiver bandwidth, were adjusted in real-time based on achieved heart rate to optimize temporal resolution while maintaining image quality.

### Pharmacological reversal and rest imaging

Following stress perfusion acquisition, aminophylline was administered intravenously to reverse the effects of regadenoson. The dose was 0.25 mg/kg for patients <40 kg, based on published paediatric protocols,^6^ or 50 mg fixed dose for patients ≥40 kg, consistent with regadenoson product literature. While reflecting established practice, this combined weight-based and fixed-dose approach results in non-linear weight-normalized dosing across the cohort, with relatively lower doses administered in smaller patients. Aminophylline was injected over 20 s, followed by 10 mL saline flush over 90 s.

After allowing at least 5 min for contrast washout and heart rate recovery, rest first-pass perfusion imaging was performed using an identical sequence following a second dose of gadolinium-based contrast agent (0.1 mL/kg, maximum 10 mL).

Late gadolinium enhancement (LGE) imaging was performed 10–15 min after the final contrast administration using phase-sensitive inversion recovery sequences. For patients with persistently elevated heart rates, LGE sequences were modified by triggering every third heartbeat to optimize image quality.

### Image analysis

Perfusion analysis was qualitative (visual assessment); semi-quantitative signal intensity analysis or quantitative perfusion mapping was not performed. Stress and rest perfusion images were reviewed in consensus by at least two experienced paediatric cardiovascular imagers. Studies were classified as positive if stress-inducible perfusion defects (subendocardial or transmural hypoenhancement present at stress but not at rest) were identified. The presence of late gadolinium enhancement and regional wall motion abnormalities were also documented. Perfusion defects at rest and/or occurring in areas of existing late gadolinium enhancement consistent were not considered stress-inducible defects.

Image quality was independently assessed by two experienced paediatric cardiovascular imagers who were blinded to regadenoson dosing (MQ 15 years experience, VM 23 years experience) using a 5-point scale: 1 = non-diagnostic, 2 = poor, 3 = adequate diagnostic quality, 4 = good, 5 = excellent. Inter-observer agreement was assessed using weighted Cohen's kappa with linear weights.

### Monitoring and safety

Continuous monitoring was maintained throughout the procedure:

- Heart rate: continuous ECG monitoring
- Non-invasive blood pressure: pre-regadenoson, during stress, post-aminophylline, and post-rest perfusion
- Oxygen saturation: continuous pulse oximetry
- Symptomatic assessment: chest pain, dyspnoea, flushing, nausea, headache, limb tingling
- Hypotension was defined as a 30% decrease in blood pressure compared to baseline and/or requiring IV fluid or inotropic support. The ≥10 mmHg blood pressure decrease used as an alternative stopping criterion during titration was distinct from this adverse event definition and was not itself classified as an adverse event.

Adverse events were classified as major (arrhythmia requiring intervention, severe bronchospasm, significant hypotension requiring vasopressor support, or necessitating study termination) or minor (hypotension resolving with aminophylline alone).

All studies were performed with a paediatric cardiologist/cardiovascular radiologist and specialist cardiac radiographers present. Resuscitation equipment was immediately available. Patients were monitored for 30–60 min following the procedure prior to discharge.

### Statistical analysis

STATA 19.5 MP (StataCorp LP, College Station, Tex) was used for statistical analysis and figures. Data were examined for normality using the Shapiro–Wilk test. Descriptive statistics are expressed as mean ± standard deviation (SD) when normally distributed and median (interquartile range [IQR]) when non-normally distributed, unless specified. Proportions are expressed as percentages. Two-tailed, independent t*-*tests were used for normally distributed data. Mann–Whitney *U* tests were used for non-normally distributed data. A Wilcoxon signed rank test was used to compare the difference in dose/weight administered (non-normally distributed) using a titration-based approach and a fixed weight dose of 8 mcg/kg or 400 mcg if >50 kg. An exploratory analysis of the relationship between age and dose/weight (mcg/kg) was assessed using linear regression analysis, with general anaesthesia as a binary covariate. Fisher’s exact test was used for categorical comparisons.

## Results

### Demographics and presence of inducible defects

Between 2015 and 2025, 31 regadenoson stress perfusion studies were scheduled for 28 unique patients (3 patients each had 2 studies) using titration-based dosing.

Two of the 31 studies could not be completed for the following reasons: one loss of ECG gating during perfusion imaging, and one cannula failure during administration of regadenoson. The remaining 29 studies were technically successful and form the basis of this analysis.

Complete dosing data from electronic prescribing records were available for 21 of 29 technically successful studies (72%). The remaining eight studies were performed early in the study period using paper-based prescribing, and these records could not be retrieved from scanned archived records prior to the implementation of the electronic record and prescribing in 2018. All eight patients with missing dosing data were awake (non-sedated) and achieved target heart rate responses.

The mean age at imaging was 11.8 ± 4 years (range 3–18 years). The mean weight was 50.4 ± 21 kg (range 14.9–91.8 kg). 7/29 (24%) of studies were performed under general anaesthesia. Nineteen of the 29 patients were male (66%).

The cohort predominately comprised studies in patients with known or suspected coronary artery disease from diverse aetiologies (*Table 1*). Kawasaki disease was the most common indication (*n* = 15, 52%), followed by transposition of the great arteries after arterial switch operation (*n* = 6, 19%), and anomalous left coronary artery from the pulmonary artery post-repair (*n* = 3, 10%).

**Table 1:** Clinical characteristics and positivity rates of patients undergoing stress perfusion studies

Patient characteristics	All (*n* = 29)	Positive (*n* = 7)
Sex M/F	19/10	4/3
Age, y	11.8 ± 4	9.0 ± 4.3
Weight	50.4 ± 21.3	35.2 ± 19.1
Dose (mcg)	160 (IQR 160–160)	160 (IQR 140–180)
Dose (mcg/kg)	3.2 (IQR 2.5–5.2)	4.6 (IQR 3.2–8)
Baseline HR (beat/min)	79 ± 13	90 ± 13
Stress HR (beat/min)	111 ± 15	119 ± 14
HR increment (beat/min)	32 ± 10	29 ± 12
HR increment (%)	42 ± 16	34 ± 15
Kawasaki disease	15 (52%)	4 (27%)
TGA post-arterial switch operation	6 (19%)	2 (33%)
ALCAPA post-repair	3 (10%)	0 (0%)
Aortic root abscess post aortic valve replacement	2 (6%)	1 (50%)
Ross-Konno procedure	1 (3%)	0 (0%)
Anomalous RCA post-repair	1 (3%)	0 (0%)
Tetralogy of Fallot with absent pulmonary valve	1 (3%)	0 (0%)

ALCAPA, anomalous left coronary artery from the pulmonary artery; RCA, right coronary artery; TGA, Transposition of the great arteries.

Stress-inducible perfusion defects were identified in 7 of 29 technically successful studies (24%): 4 patients with Kawasaki disease (27% of Kawasaki cohort), 2 with TGA post-arterial switch operation (33% of TGA cohort), and 1 following aortic root surgery with coronary artery reimplantation.

Perfusion abnormalities were conservatively managed in 6/7 patients. One patient underwent percutaneous coronary intervention to the right coronary artery with a drug-eluting stent.

### Adequacy of HR response

The mean HR at rest was 79 ± 13 bpm, increasing to 111 ± 15 bpm at stress. The mean increase in HR following regadenoson was 32 ± 10 bpm (range 16–56 bpm), representing a percentage increase of 42 ± 16% (range 16–77%).

A priori, a target heart rate threshold of ≥20% was selected for 28 of 29 studies. In one patient with baseline tachycardia (HR >110 bpm), the target HR threshold was reduced to ≥15% at clinician's discretion to avoid excessive tachycardia and the need for protocol modifications that might compromise image quality. The target HR threshold was met in all studies, with the patient with a lower target HR achieving a 16% increase (weight >50 kg, dose administered 4.6 mcg/kg). A stress-inducible perfusion defect was identified in this patient.

There was no significant difference in percentage HR increment in patients who had a positive stress perfusion test compared to those who were negative (34 ± 15% vs. 44 ± 17%, *P* = 0.14).

### Regadenoson dose

**Figure 1:**
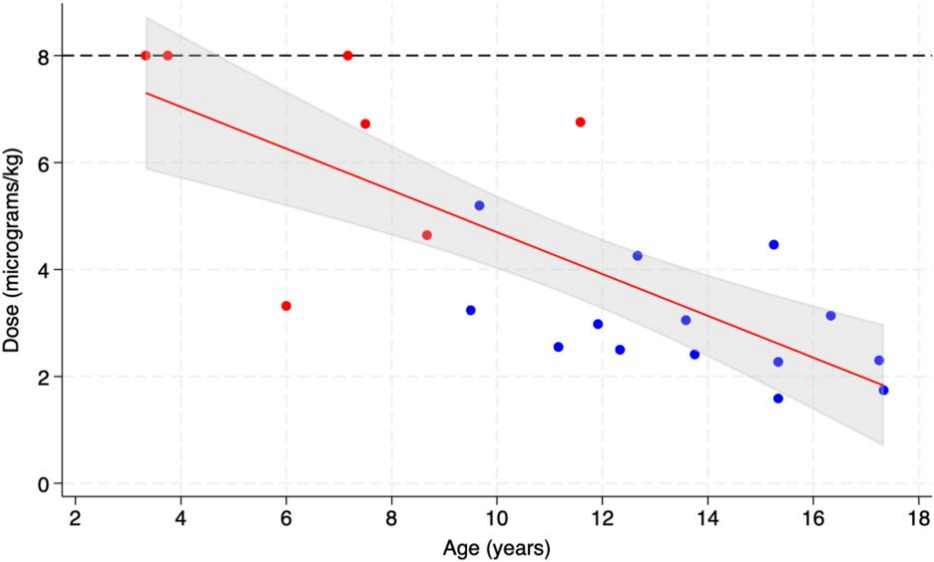
Scatter plot of dose (regadenoson micrograms/kg) vs. age (years). Blue dots (unsedated), Red dots (general anaesthesia). The figure shows a linear regression line (red) with 95% confidence interval (light grey). Dashed line 8 mcg/kg, conventional weight-based dosing.

Weight-specific dose administration was only available from electronic records for 21/29 patients. The median regadenoson dose/kg was 3.2 mcg/kg (IQR 2.5–5.2 mcg/kg, range 1.6–8 mcg/kg). This represents a 60% dose reduction at the group level compared to weight-based dosing (8 mcg/kg or 400 mcg if >50 kg) protocol (one-sample Wilcoxon signed-rank test, z = −3.9, *P* < 0.0001), *Figure 1*.

**Figure 2:**
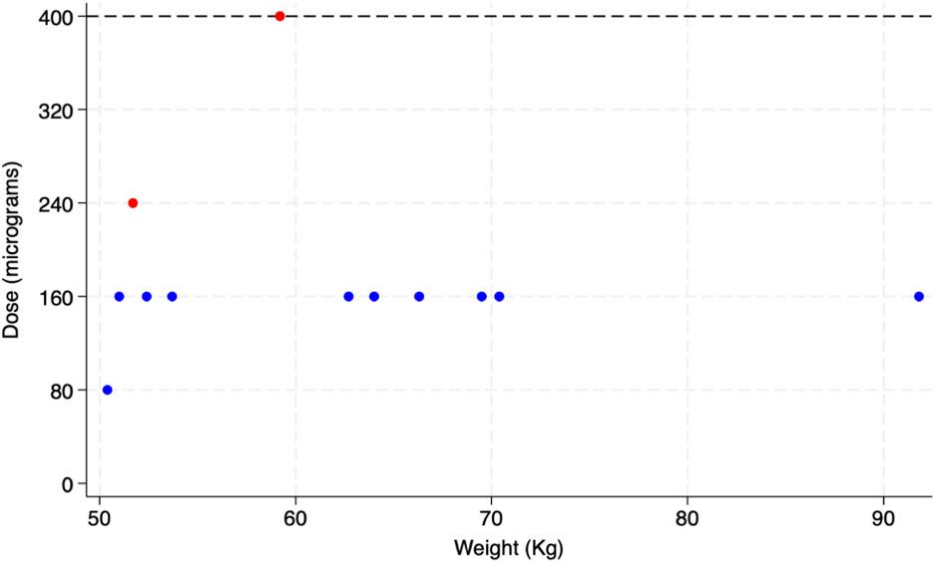
Scatter plot of dose (regadenoson micrograms) vs. weight (kg) in patients with a weight greater than 50Kg. Blue dots (unsedated), red dots (general anaesthesia). Dashed line 400 µg, conventional dose for children >50 kg.

12/21 studies were performed in patients >50 kg and in this group, the median dose of regadenoson required to achieve target HR was 2.5 mcg/kg (IQR 2.3–3.1 mcg/kg). A dose of less than or equal to160micrograms was administered in 10/12 patients (all unsedated patients), *Figure 2*.

There was no difference in dose/weight in patients who had a positive stress perfusion test compared to those who were negative (4.6 [IQR 3.2–8] mcg/kg vs. 3.0 [IQR 2.4–4.5] mcg/kg, *P* = 0.09).

### Sensitivity analysis for missing data

There were no significant differences in age, (14.1 ± 3.1vs 11.4 ± 4.2 years, *P* = 0.1), weight (56.0 ± 20.6 vs. 46.8 ± 20.9 kg, *P* = 0.3), image quality scores (median 4 vs. 4, *P* = 0.6), or heart rate increment (41 ± 16% vs. 42 ± 17%, *P* = 0.9). No patient with a positive stress perfusion study had missing data, (0% vs. 33%, *P* = 0.08). Notably, no patients with missing dose data underwent general anaesthesia.

To assess the potential impact of missing dosing data, we performed a sensitivity analysis under a worst-case assumption: that all eight patients with missing data received maximum doses (8 mcg/kg for patients <50 kg, or 400 mcg for patients ≥50 kg). Under this conservative scenario, the median dose across all 29 patients would be 4.6 mcg/kg, which still represents a 42.5% reduction compared with standard 8 mcg/kg protocols (Wilcoxon signed-rank test, z = −4.4, *P* < 0.0001). This suggests that even if missing cases systematically required higher doses, substantial dose reduction would remain evident.

### Blood pressure response

Blood pressure was monitored before and during stress in all patients. The systolic BP at rest was 111 ± 20 mmHg, decreasing to 108 ± 23 mmHg during stress. The diastolic BP at rest was 57 ± 13 mmHg, decreasing to 52 ± 14 mmHg during stress. The mean arterial pressure (MAP) at rest was 74 ± 15 mmHg, decreasing to 70 ± 17 mmHg during stress.

**Figure 3:**
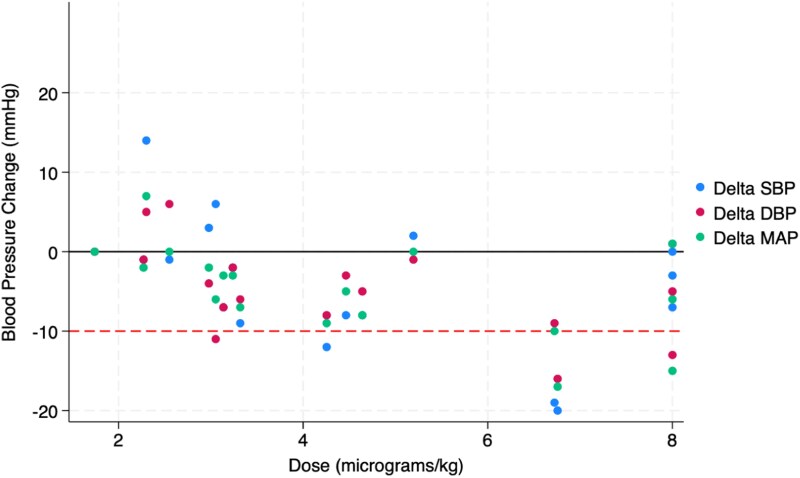
Scatter plot of blood pressure change (mmHg) vs. dose (regadenoson micrograms. Blue dots (systolic BP change), red dots (diastolic BP change), green dots (mean arterial pressure change). Dashed red reference line at −10 mmHg.

Nine patients (31%) demonstrated a blood pressure decrease of >10 mmHg in at least one parameter (systolic or mean arterial pressure) following regadenoson administration, meeting alternative criteria for adequate pharmacological stress. Among these nine patients, complete dosing data were available for five, who received a mean dose of 5.8 ± 2.0 mcg/kg (range 3.1–8.0 mcg/kg), *Figure 3*. Three patients undergoing general anaesthesia (42%) had blood pressure decreases of >10 mmHg.

All nine patients with significant blood pressure decreases also achieved the target heart rate response, indicating that both haemodynamic markers of adequate stress were present. There was no association between blood pressure decrease >10 mmHg and presence of stress-inducible perfusion defects (*P* = 0.8), suggesting that blood pressure response did not confound diagnostic sensitivity.

### Relationship between dose and age and sex

Among the 21 patients with complete dosing data, there was a strong inverse correlation between age and dose in unadjusted analysis (*r* = −0.79, *P* < 0.001), with younger children receiving significantly higher doses than older children. However, general anaesthesia was used more commonly in younger children, creating confounding between age and anaesthesia status. After adjusting for anaesthesia type in a multivariable regression model the age effect was no longer statistically significant (*P* = 0.1). This suggests that the apparent relationship between younger age and higher doses is largely explained by the differential use of general anaesthesia in younger children rather than age-dependent pharmacological differences, but this analysis is limited by small numbers.

There were no differences in dose/weight between male and female patients (3.3 [IQR 2.5–6.0] mcg/kg vs. 3.1 [IQR 2.5–4.5] mcg/kg, *P* = 0.9). There were no differences in percentage HR increment between male and female patients (41 ± 18% vs. 44 ± 14%, *P* = 0.7).

### Safety and complications

No major complications were observed in patients undergoing regadenoson stress perfusion imaging. One patient reported mild palpitations during stress.

No patient had symptomatic hypotension or required additional pharmacological haemodynamic support (e.g. phenylephrine) or intravenous fluid boluses to maintain blood pressure after the administration of regadenoson. All patients had normalization of blood pressure following administration of aminophylline.

No post-procedural ECG abnormalities were detected in patients. No patient had regadenoson associated bronchospasm.

### Image quality

**Figure 4:**
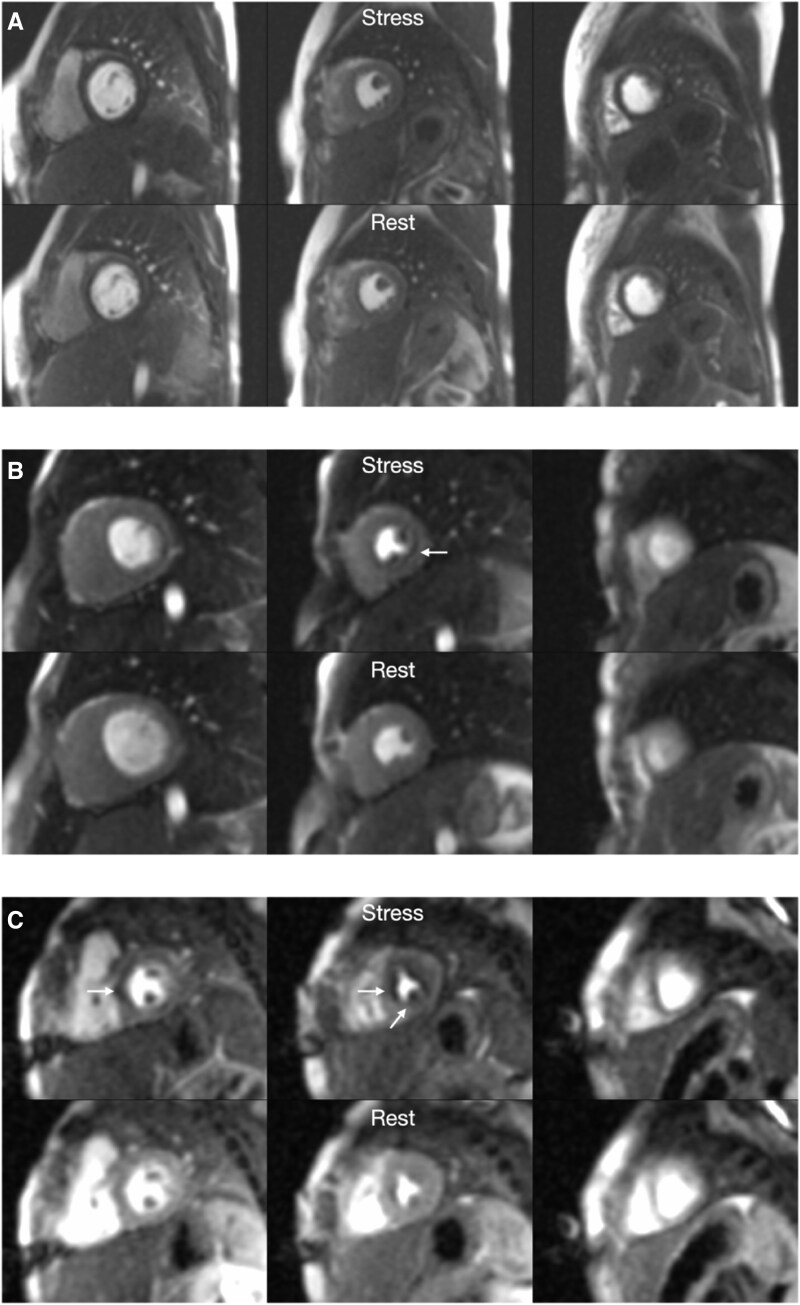
Cardiac stress perfusion imaging studies. Each panel displays short-axis slices (base, mid, and apical) arranged left to right, with stress perfusion images in the top row and rest perfusion images in the bottom row. *(A*) Normal perfusion study in a 12-year-old patient (64 kg). Regadenoson dose: 160 mcg. Heart rate increased 40% (75→105 bpm). Image quality: 5/5. *(B*) Abnormal stress perfusion study showing perfusion defect in the mid-LV inferolateral segment in a 12.7-year-old patient (38 kg). Regadenoson dose: 160 mcg. Heart rate increased 50% (80→120 bpm). Image quality: 4.5/5. *(C*) Abnormal stress perfusion study showing perfusion defects in the basal and mid-LV inferolateral segments and posteromedial papillary muscle in a 7.2-year-old patient (22 kg). Patient subsequently underwent PCI for proximal RCA stenosis. Regadenoson dose: 180 mcg (8 mcg/kg) administered under general anaesthesia. Heart rate increased 30% (76→98 bpm). Image quality: 3.5/5.

All 29 technically successful studies were independently scored for image quality by two experienced observers, *Figure 4*.

Observer 1: Median 4 (IQR 4–4, range 3–5). Observer 2: Median 4 (IQR 4–5, range 3–5). Inter-observer agreement was moderate with weighted Cohen's kappa = 0.54 (SE 0.13, *P* < 0.001) and 86% weighted agreement. All disagreements were within one point on the ordinal scale, and no studies were rated non-diagnostic (score <3) by either observer.

There were no associations between disagreements and study-positivity, imaging-type (SSFP vs. Gradient echo), or HR-increment. There was a non-significant trend for the presence of 1point of disagreement in the assessment of image quality in GA cases compared to unsedated studies (*P* = 0.07). All 29 studies (100%) were rated as diagnostic quality (score ≥3) by both observers. Twenty-five studies (86%) were rated as good or excellent quality (score ≥4) by at least one observer, with 19 studies (66%) rated as good quality or better by both observers. No study was excluded based on an inadequate imaging score (<3).

No studies required repeat imaging due to inadequate image quality. Excessive tachycardia resulting in image acquisition every other heartbeat occurred in only 1 patient.

## Discussion

This single-centre retrospective analysis demonstrates that heart rate-targeted titration of regadenoson for paediatric stress perfusion CMR is feasible and can achieve adequate pharmacological stress with substantially lower drug doses than published weight-based protocols and without major adverse events. The median dose of 3.2 mcg/kg represents a 60% reduction compared to the standard 8 mcg/kg (400 mcg >50 kg) regimen. This includes children >50 kg, where the most commonly administered dose was 160 µg, also representing a 60% reduction compared to the standard 400 µg dose. Target heart rate response was achieved in all 29 technically successful studies without major adverse events, confirming the feasibility of this individualized approach. These data support the need for further pharmacokinetic studies on regadenoson hyperaemia dosing in children.

### Adequacy of pharmacological stress

A critical question raised by dose titration is whether reduced drug exposure achieves adequate coronary hyperaemia for diagnostic sensitivity. Direct measurement of myocardial blood flow was not performed; however, this limitation applies equally to fixed weight-based protocols, which also assume adequate hyperaemia. Within these constraints, several observations (as described below) support the hypothesis that our titration protocol delivered adequate pharmacological stress for diagnostic sensitivity, while highlighting areas requiring formal validation.

First, while our protocol targeted a minimum 20% heart rate increase, the median achieved response was 42% (range 16–77%), comparable to published weight-based paediatric protocols (41–49%, *Table 2*). For typical paediatric baseline heart rates of 70–90 bpm, a 20% threshold translates to 14–18 bpm increases, exceeding the >10 bpm increment considered adequate for pharmacological stress in adult practice.^12^ This increment is the same as our institution's historical adenosine stress perfusion protocol, which was validated against coronary angiography.^13^ Additionally, 31% of patients demonstrated blood pressure decreases >10 mmHg, meeting alternative SCMR consensus criteria for adequate vasodilatory response.^12^ The incorporation of multiple physiological markers (heart rate, blood pressure, symptoms) provides broader evidence of pharmacological effect than reliance on heart rate alone. Importantly, the single patient requiring protocol modification to a 15% threshold due to baseline tachycardia demonstrated inducible ischaemia, confirming adequate stress despite the lower threshold.

**Table 2:** Published studies of paediatric regadenoson use

Study (year)	Journal	Cohort/extract	centre	*n*	Age (mean/median and range)	Weight (mean/median and range)	Regadenoson dosing	HR increase—metric	%HR increase	Positivity rate (inducible)
Noel et al., 2017^3^	Pediatr Radiol (2017) 47:280–289	Paediatric/young adult pts	TC	31	15.8 ± 1.7 year (12–22)	60 ± 15 kg	400 mcg fixed dose	Baseline 72 ± 14 → peak 124 ± 17 bpm (Δ95 ± 50%)	95% (SD 50)	4/31 (12.9%)
Noel et al., 2018^4^	Pediatr Cardiol (2018) 39:1249–1257	Arterial Switch Operation (paediatric/young adult)	TC	36	15.1 ± 4.5 year (0.2–22)	61.6 ± 21.5 kg (3.8–93)	400 mcg fixed dose	Baseline 72 ± 13 → peak 120 ± 17 bpm (Δ95% ± 50%)	95% (SD 50)	7/36 (19.0%)
Wilkinson et al., 2019^6^	Radiol Cardiothorac Imaging (2019) 31: e190061	Infants/young children <40 kg	TC	46	Median 9.0 year (2 mo–13.9 yr)	Median 27.6 kg (range 3.8–39.3)	8 mcg/kg	Baseline 87 ± 16 → peak 127 ± 14 bpm (Δ49% ± 23%)	49% (SD 23)	5/46 (10.8%)
Doan et al., 2019^5^	Am J Cardiol (2019) 124(7):1125–1132	Kawasaki Disease	TC	41	Median 11.2 years (range 2.2 to 18.6)	weight 41 kg (range 13 to 93.4)	8 mcg/kg	Baseline 78 ± 15 → peak 126 ± 16 bpm (Δ48% ± 13%)	48% (SD 13)	14/41 (34%)
Schloss et al., 2021^8^	Cardiol Res. 2021 8;12(6):329–334	GA paediatric	NWCH	8	Mean 4.2 year (2–6.2)	Mean 18.5 kg (10–30.5)	8 mcg/kg (max 400 mcg)	Baseline 99 ± 19 → peak 122 ± 15 bpm (Δ23%)	23% (Estimated SD 18)	1/08 (12.5%)
Husain et al., 2021^9^	J Cardiovasc Magn Reson. 2021 22;23:135	Coronary Allograft Vasculopathy	LC	26	Mean 16.3 ± 3.1	Mean 62.4 ± 20.8kg	6–10 mcg/kg (smaller pts), up to 400 mcg (larger)	Baseline 86 ± 9 → stress not reported	N/A	6/24 (25%)
Patel et al., 2022^10^	Children 2022, 9(9), 1332	Mixed paediatric	LC	38	Median 15 years (IQR 12–17)	Median 61 kg (IQR 51–75)	6–10 mcg/kg (smaller pts), up to 400 mcg (larger)	Baseline 86 ± 17 → peak 118 ± 18 bpm (Δ41% ± 27%)	41% (SD 27)	4/38 (10.5%)
**Quail et al., 2026**	**Current**	**Mixed paediatric**	**GOSH**	**29**	**Mean 11.8 years**	**Median weight 51.7 kg (range 14.9–91.8)**	**3.2 (IQR 2.5–5.2) mcg/kg**	**Baseline 79** **±** **13 → peak 112** **±** **15 bpm (Δ42%** **±** **16%)**	**42% (SD 16)**	**7/29 (24.0%)**

Studies from the same centre may include duplicated patient data across publications. LC: Lurie Children’s Hospital of Chicago, USA; NWCH: Nationwide Children's Hospital, Columbus, OH, USA; GOSH: Great Ormond Street Hospital for Children, London, UK; TC: Texas Children’s Hospital, Houston, TX, USA;

Second, patients with positive studies achieved similar heart rate responses to those with negative studies (34 ± 15% vs. 44 ± 17%, *P* = 0.14), demonstrating that inducible ischaemia was detected across the range of haemodynamic responses and was not confined to patients receiving higher doses.

Third, the 24% positivity rate in our high-risk cohort (31% in Kawasaki disease, 33% in post-arterial switch patients) suggests appropriate detection of clinically significant coronary disease and not lower than the reported in published paediatric regadenoson mixed pathology series employing weight-based dosing (10.5–12% *Table 2*). For patients with negative studies, whether higher doses would have unmasked additional perfusion defects cannot be determined from this data, though this uncertainty applies to all dosing strategies.

Importantly, fixed weight-based dosing regimens may expose a subset of patients to unnecessarily high drug doses and excessive haemodynamic stress. In two of the earliest and largest published paediatric series employing standard 400 mcg fixed dosing, mean heart rate increases were 95% with standard deviations of ±50% [Noel 2017, Noel 2018].^3,4^ This implies that approximately one-sixth of patients experienced heart rate increases exceeding 145%. It is noteworthy that in these studies, patients were older and had a mean body weight of >60 kg. Such excessive tachycardia creates several issues. First, very high heart rates (>140–150 bpm) necessitate sequence modifications including reduced spatial resolution, decreased slice coverage, and increased bandwidth to maintain temporal resolution, potentially compromising diagnostic image quality. In fact, Noel *et al*.^3^ reported that excessive tachycardia resulted in acquisitions exceeding RR interval image acquisition settings in 26% of patients (compared to 1/29 [3%] in this study). Secondly, shortened diastolic periods reduce myocardial perfusion time and may paradoxically impair contrast kinetics. Finally, dose-dependent adverse events, including dyspnoea and hypotension, are more frequent and severe at higher drug exposures, which may be particularly poorly tolerated in patients with compromised ventricular function or complex congenital heart disease.

In contrast, our titration approach achieved a more consistent haemodynamic response with substantially reduced variability (mean 42% ± 16% increase), suggesting better individualization of drug exposure to physiological response. By ceasing drug administration once an adequate threshold was achieved, titration avoided exposing drug-sensitive individuals to unnecessarily high doses whilst ensuring adequate stress in those requiring higher cumulative amounts.

### Influence of age and anaesthesia on dose requirements

The confounding between age and anaesthesia status in our cohort highlights the complexity of interpreting dose requirements in paediatric populations. While younger children appeared to require higher weight-normalized doses, after adjusting for anaesthesia type, the age effect became non-significant. This suggests that the apparent age effect is largely explained by the increased use of general anaesthesia in younger children rather than intrinsic age-related pharmacological differences. This observation has important practical implications: clinicians should anticipate higher dose requirements in anaesthetized patients irrespective of age, rather than assuming younger children *per se* are inherently less sensitive to regadenoson.

The substantial dose reductions achieved through titration (median 3.2 mcg/kg vs. standard 8 mcg/kg, representing 60% reduction) suggest potential cost savings. However, realizing these savings in clinical practice faces practical constraints. Regadenoson is supplied in single 400 mcg/5 mL vials (UK: ∼£68), and aseptic pharmaceutical regulations typically preclude sharing vials between patients, limiting opportunities for cost recovery through multi-patient use. For low-volume centres, such as ours, performing approximately 5 paediatric stress studies annually, the economic impact is modest: potential theoretical savings of approximately £150 annually, even if partial vials could be credited, insufficient to justify protocol adoption solely on cost grounds.

### Refinement of paediatric dosing strategies

Our titration approach, while achieving substantial dose reductions, targets heart rate response rather than direct pharmacokinetic or pharmacodynamic endpoints. Further refinement of paediatric regadenoson dosing requires formal pharmacokinetic data to characterize drug exposure, clearance, and dose-response relationships across paediatric age ranges. Until formal pharmacokinetic studies establish optimal weight-based dosing or validation studies confirm the safety and efficacy of titration protocols, centres considering response-guided approaches should implement prospective monitoring of doses, heart rate responses, adverse events, and positivity rate to establish institutional experience.

Future prospective validation could incorporate quantitative myocardial blood flow (MBF) or coronary sinus flow measurement to confirm adequate coronary hyperaemia with titrated dosing. Published adult data suggest that stress MBF >1.4 mL/g/min reliably indicates adequate hyperaemia.^14^ A prospective observational study could determine whether titrated regadenoson doses achieve stress MBF above this threshold, thereby directly validating the physiological adequacy of this approach. Such a study would also provide the first paediatric MBF reference data for regadenoson stress. Given the low frequency of paediatric stress perfusion studies at individual institutions, multicentre collaboration would be essential to achieve an adequate sample size.

### Study limitations

This retrospective single-centre study has important limitations. Heart rate response, blood pressure changes, and clinical symptoms are indirect markers of pharmacological effect and may not reliably reflect coronary vasodilation. Without quantitative perfusion measurements (e.g. myocardial blood flow quantification), we cannot definitively determine whether our titration approach produced adequate coronary vasodilation for diagnostic sensitivity. However, current paediatric weight-based protocols (8 mcg/kg) were also extrapolated from adult studies and similarly lack quantitative perfusion validation. Thus, both approaches require formal validation using quantitative perfusion techniques.

All regadenoson studies at our institution used the titration protocol; therefore, direct comparison with fixed-dose regadenoson within this cohort was not possible. Comparisons with published weight-based series are descriptive and cannot establish superiority.

Complete dosing data were available for 21 of 29 studies (72%). Missing dosing data from 8 patients (all non-sedated, from the early study period with paper prescribing) may bias our dose calculations. However, sensitivity analysis assuming maximum doses in these patients still demonstrated a 42.5% dose reduction (*P* < 0.0001), suggesting our findings are robust to this missing data. Additionally, comparison of demographics and outcomes between groups showed no significant differences, supporting the robustness of our findings.

Aminophylline was administered routinely to expedite heart rate recovery prior to rest imaging. Whilst reversal of regadenoson-induced hyperaemia is incomplete at 5 min, diagnostic interpretation incorporated both rest perfusion imaging and late gadolinium enhancement, the latter performed 10–15 min post-contrast when residual hyperaemic effects are minimal.^15^

The added complexity of titration (3–5 min additional procedure time, requirement for experienced operators) must be weighed against the practical advantages of regadenoson over adenosine infusion in paediatric settings. For centres with established adenosine protocols, the incremental benefit of regadenoson titration may not justify protocol modification.

The modest sample size limits statistical power for subgroup analyses and precludes robust conclusions about factors predicting dose requirements. The cohort consisted predominantly of Kawasaki disease (52%), post-arterial switch TGA (19%), and post-repair ALCAPA (10%), and applicability to other populations warrants evaluation. The protocol was implemented at a tertiary paediatric cardiac centre with experienced operators directly supervising dosing and requires real-time clinical decision-making, adding 3–5 min to procedure time.

## Conclusion

This single-centre study demonstrates that heart rate-targeted regadenoson titration is feasible and achieves lower drug exposure than published weight-based protocols (median 3.2 mcg/kg vs. 8 mcg/kg) without significant adverse events. The 24% positivity rate in this high-risk cohort and haemodynamic responses suggest adequate stress was achieved, but prospective validation with myocardial blood flow quantification would be required to confirm that adequate hyperaemia has been achieved. This is now feasible in prospective studies as improvements in quantitative perfusion technologies make it more applicable to the paediatric population.

Whether individualized titration offers advantages over fixed dosing or adenosine protocols requires prospective comparison, but these findings may inform future pharmacokinetic studies and dosing algorithm development for paediatric stress perfusion CMR.

## Lead author biography


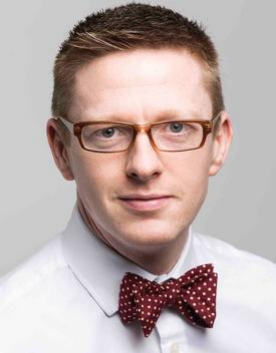


Dr Michael Quail is a consultant paediatric cardiologist at Great Ormond Street Hospital for Children and Honorary Associate Professor at University College London, UK. His research focuses on advanced cardiovascular imaging in congenital heart disease, with research interests in stress perfusion CMR and cardiovascular physiology. He is funded by a British Heart Foundation Intermediate Clinical Research Fellowship.

## Data Availability

The datasets generated and analysed during this study are available from the corresponding author on reasonable request, subject to institutional data governance requirements.
